# Including Eye Movement in the Assessment of Physical Fatigue Under Different Loading Types and Road Slopes

**DOI:** 10.3390/jemr19010013

**Published:** 2026-01-27

**Authors:** Yixuan Wei, Xueli Wen, Shu Wang, Lanyun Zhang, Jianwu Chen, Longzhe Jin

**Affiliations:** 1 State Key Laboratory of Metal Mine Mining Safety and Disaster Prevention and Control, University of Science and Technology Beijing, Beijing 100083, China; weiyixuan@ustb.edu.cn (Y.W.);; 2 School of Resources and Safety Engineering, University of Science and Technology Beijing, Beijing 100083, China; 3 Research Institute of Macro-Safety Science, University of Science and Technology Beijing, Beijing 100083, China; 4 NHC Key Laboratory for Engineering Control of Dust Hazard, University of Science and Technology Beijing, Beijing 100083, China; 5 Industrial Design, College of Mechanical & Electrical Engineering, Nanjing University of Aeronautics and Astronautics, Nanjing 210016, China

**Keywords:** emergency rescuer, loading type, road slope, eye movement metrics, physical fatigue

## Abstract

**Background**: Emergency rescuers frequently carry heavy equipment for extended periods, making musculoskeletal disorders a major occupational concern. Loading type and road slope play important roles in inducing physical fatigue; however, the assessment of physical fatigue under these conditions remains limited. **Aim**: This study aims to investigate physical fatigue under different loading types and road slope conditions using both electromyography (EMG) and eye movement metrics. In particular, this work focuses on eye movement metrics as a non-contact data source in comparison with EMG, which remains largely unexplored for physical fatigue assessment. **Method**: Prolonged load-bearing walking was simulated to replicate the physical demands experienced by emergency rescuers. Eighteen male participants completed experimental trials incorporating four loading types and three road slope conditions. **Results**: (1) Loading type and road slope significantly affected EMG activity, eye movement metrics, and perceptual responses. (2) Saccade time (ST), saccade speed (SS), and saccade amplitude (SA) exhibited significant differences in their rates of change across three stages defined by perceptual fatigue. ST, SS, and SA showed strong correlations with subjective fatigue throughout the entire load-bearing walking process, whereas pupil diameter demonstrated only a moderate correlation with subjective ratings. (3) Eye movement metrics were incorporated into multivariate quadratic regression models to quantify physical fatigue under different loading types and road slope conditions. **Conclusions**: These findings enhance the understanding of physical fatigue mechanisms by demonstrating the potential of eye movement metrics as non-invasive indicators for multidimensional fatigue monitoring in work environments involving varying loading types and road slopes.

## 1. Introduction

According to statistics from the National Emergency Rescue Center of China, approximately 88,000 emergency rescue personnel are currently employed in the mining and hazardous chemical sectors, where they play a critical role in safeguarding public health and property [1]. Rescuers are required to carry heavy equipment for prolonged periods during both routine training and operational missions. This sustained physical burden substantially elevates the risk of injuries to the back, waist, and shoulders. Consequently, musculoskeletal disorders have emerged as a significant occupational health and safety concern among rescuers and related personnel [2,3].

Prolonged load-bearing tasks are a primary cause of muscle fatigue, which is commonly defined as an exercise-induced decline in the capacity to generate force [4]. Multiple factors influence the development of physical fatigue during load-bearing activities. Existing studies have documented the biomechanics associated with different load distributions, such as symmetric and asymmetric carrying [5,6,7]. The effects of terrain on postural stability have also been analyzed [8,9,10]. However, previous research has often overlooked the effects of load type and road slope on fatigue accumulation and the assessment of emergency rescuers.

Physical fatigue can be assessed using various approaches, including subjective scale-based methods, bio-signal measurement methods, image recognition methods, and modeling-based methods [11]. Muscle fatigue is typically assessed through electromyography (EMG), with non-invasive surface EMG signals widely regarded as an objective indicator of muscular fatigue. Numerous studies have employed the median frequency (MF) of EMG signals as a key parameter [12,13,14,15]. Beyond spectral features, signal complexity metrics, such as those derived from recurrence plots, entropy measures, or fractal analysis, have also been shown to indicate muscle fatigue [16,17,18,19]. In addition, a variety of classification algorithms have been employed to distinguish fatigued from non-fatigued muscle states, including random forests, support vector machines, naïve Bayes classifiers, and convolutional neural networks [20,21,22]. EMG-based fatigue monitoring has been applied in occupational and clinical contexts such as construction work, surgical operations, and manual handling of heavy loads [23,24,25]. However, EMG measurement requires electrodes to be placed directly on the skin over the target muscle group, which may compromise operator comfort and limit its suitability for long-term monitoring of physical fatigue in practical environments.

In complex occupational environments, one of the most occurred progressions from fatigue to safety risk can often be conceptualized as: physical fatigue causes cognitive failure and further leads to risk-related outcomes [26]. Physical fatigue impairs workers’ capacity to observe and accurately interpret environmental cues, thereby increasing susceptibility to accidents [27]. In addition to muscle fatigue assessment, recent research has increasingly employed electroencephalography (EEG), eye-tracking, and electrocardiography (ECG) to detect both mental and physical fatigue in safety-critical contexts [28,29,30]. For instance, eye movements such as saccades, blinks, and pupil size have been correlated with prolonged mental fatigue in driving tasks [31]. Similarly, Stasia et al. [32] demonstrated that peak saccadic velocity serves as a sensitive indicator of mental fatigue. In another domain, the fatigue and wakefulness states of tower crane drivers can be derived by using the combinations of EEG measures and the Karolinska Sleepiness Scale (KSS). When integrated with a multi-scale attention convolutional neural network, classification accuracy reached 98.7% across ten participants [33]. Physiological signals such as ECG and photoplethysmography (PPG) have also been utilized to assess fatigue, with heart rate variability (HRV) identified as a particularly robust indicator [34,35,36,37,38]. Across the literature, findings consistently show that reliance on a single physiological indicator is vulnerable to factors such as environmental conditions, the operators’ emotional state, and task demands. In contrast, multidimensional measurement approaches, integrating multiple physiological signals, have achieved fatigue detection accuracies exceeding 94%, highlighting their superior robustness for fatigue monitoring and risk prevention [38].

Despite extensive research on fatigue detection, relatively few studies have examined the detection of workers’ physical fatigue through eye movement parameters. Compared to electromyography (EMG), eye movement signals provide a non-contact and less intrusive measurement approach. In earlier years, eye gaze parameters were used to assess attention allocation and cognitive fatigue [39,40]. Recently, eye-tracking technology has been widely used in safety-related research issues, including hazard detection [28,41,42], visual attention [43,44], and cognitive load assessment [45,46,47]. However, validity and applicability of eye movement signals for detecting physical fatigue remain to be systematically verified.

In this study, a simulated task of prolonged load-bearing walking was conducted to replicate the physical demands of emergency rescue personnel. During the experiment, participants’ eye movement signals were recorded in parallel with electromyographic data and subjective self-assessments, enabling an integrated analysis of fatigue-related changes. This study aims to investigate the assessment of physical fatigue under different loading types and road slope conditions using EMG and eye movement metrics, with a particular focus on the integration of eye movement measures. Three research questions are addressed:

**RQ1:**  Do loading type and road slope significantly influence EMG activity, eye movement metrics, and perceptual responses? If so, how? Accordingly, three hypotheses are proposed:

**H1(a,b).**  *(a) Loading type and (b) road slope significantly influence surface EMG signals.*

**H2(a,b).**  *(a) Loading type and (b) road slope significantly influence eye movement metrics.*

**H3(a,b).**  *(a) Loading type and (b) road slope significantly influence perceptual metrics.*

**RQ2:**  Do eye movement metrics have the potential to reflect physical fatigue? Specifically, three hypotheses are proposed:

**H4.**  *Eye movement metrics exhibit significant changes across three phases defined by perceptual fatigue.*

**H5.**  *Eye movement metrics are significantly correlated with perceptual fatigue levels across the entire load-bearing walking process.*

**H6.**  *Changes in eye movement metrics are significantly correlated with changes in EMG activity and overall physical fatigue.*

**RQ3:**  How can eye movement metrics contribute to the assessment of physical fatigue under different loading types and road slope conditions when combined with electromyographic measures?

The contributions of this study are three-fold: (1) understanding how loading types and road slopes affect people’s EMG, eye movement, and subjective measures; (2) advancing the theoretical understanding of fatigue by revealing the potential of eye movement signals as non-invasive indicators when combined with EMG measures; and (3) offering practical insights for developing multidimensional fatigue monitoring models to improve safety and performance in load-bearing work environments.

## 2. Material and Methods

### 2.1. Participants

The aim of this experiment was to simulate the long-term, load-bearing walking procedure of workers in deep mines or emergency rescue teams. A total of 18 healthy male participants with no musculoskeletal diseases voluntarily participated in this study, ensuring a comparable baseline health status. All participants had their body fat percentage measured using an MA8000 body composition analyzer (Charder Electronic Co., Ltd., Taiwan, China) to exclude individuals with obesity and ensure comparable anthropometric characteristics across the sample. It should be noted that all participants were required to wear eye-tracking glasses during the experiment, thus ensuring that all participants had normal vision and were corrected to normal vision. Furthermore, the participants had no current use of prescription medications, habitual alcohol or tobacco consumption, or other detrimental habits. In this study, all participants were “no habitual alcohol consumers”, which was defined as (1) no clinical history of alcohol dependence and (2) an alcohol intake frequency of less than twice a week with no more than 2 standard drinks per occasion. Demographic information of the participants is listed as following: 22.37 ± 1.79 years old, 177 ± 4.55 cm, 69.02 ± 6.85 kg, 21.93 ± 1.43 kg/m^2^, and body fat percentage 17.32 ± 4.95%.

We adopted G*Power 3.1 tool to ensure that our sample size is adequate for the statistical methods applied in this study. According to the calculation, a sample size of 15 participants was required to achieve an effect size of 1, with a significance level of 0.05 and a statistical power of 0.80. Therefore, a sample size of 18 participants was sufficient for our experiment.

### 2.2. Experiment Design

**Figure 1:**
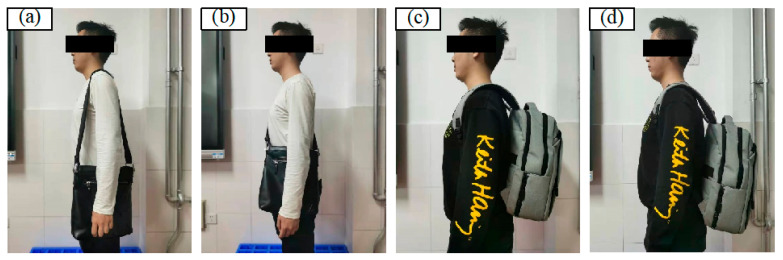
Four loading types. (**a**) Single-sided loading. (**b**) Cross-body loading. (**c**) High-position bilateral shoulder loading. (**d**) Low-position bilateral shoulder loading.

The present study evaluated the development of muscle fatigue during constant load-walking exercises. Previous research has found that male adults, lacking training, should refrain from carrying materials weighing in excess of 15% of their body weight over extended periods [48,49]. In this study, the weighing was set at 10.0 kg with the objective of guaranteeing the safety of the participants. The experiment consisted of a series of independent trails to investigate how combinations of load type and road slope could induce fatigue. Four loading types were tested in this study, including single-sided loading (Type A), cross-body loading (Type B), high-position bilateral shoulder loading (Type C), and low-position bilateral shoulder loading (Type D); see Figure 1. The road slope was configured to represent three distinct conditions: uphill, downhill, and flat. The incline of each condition was set at an angle of 10°. Overall, a 4 (loading type) × 3 (road slope) study was conducted; see Table 1. The dependent variables measured in this study were electromyography signals, subjective fatigue evaluation, and eye-tracking parameters.

To minimize the influence of sunlight and ambient illumination, curtains were drawn throughout the experiment. The room was lit by two LED panel lights with a total illuminance of 350 lumens, positioned on the ceiling behind the participant’s head, ensuring a uniform visual field. Participants faced a white wall, providing a consistent background across all walking conditions.

### 2.3. Equipment

**Figure 2:**
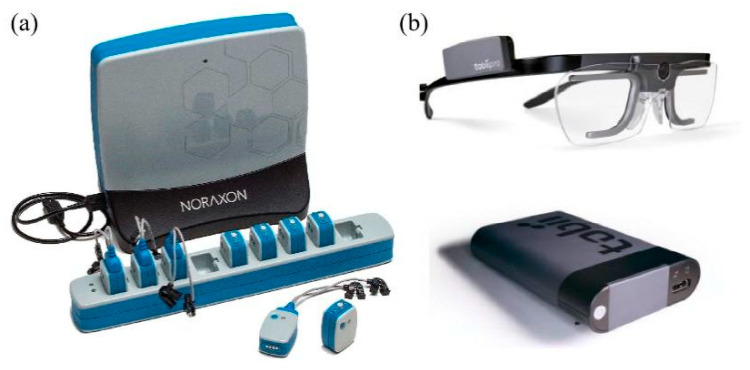
Experiment equipment. (**a**) Noraxon’s Ultium EMS sensor system. (**b**) Tobii Pro Glass2.

Surface electromyogram (EMG) signals were measured and collected by three pairs of disposable electrodes (Ag/AgCl, size: 50 mm diameter, Shanghai Shenfeng Co., Shanghai, China) and Noraxon’s Ultium EMS sensor system (Noraxon USA, Inc., Scottsdale, Arizona, USA), which provided a non-invasive measurement of muscles [50]; see Figure 2a. The recorded signals are sampled to 1000 samples per second. Furthermore, the Tobii Pro Glass 2 [51] was used to effectively measure and record the participants’ eye movement behaviors during the experiment; see Figure 2b. Tobii Pro Glass 2 utilized a sampling frequency of 100 Hz, incorporating a range of eye movement tracking functions. These functions encompassed the corneal reflex, binocular acquisition, and dark pupil tracking, enabling the identification of various eye movement behaviors, such as fixation, saccades, and blinks.

### 2.4. Dependent Variables

#### 2.4.1. Surface EMG Measurement

**Figure 3:**
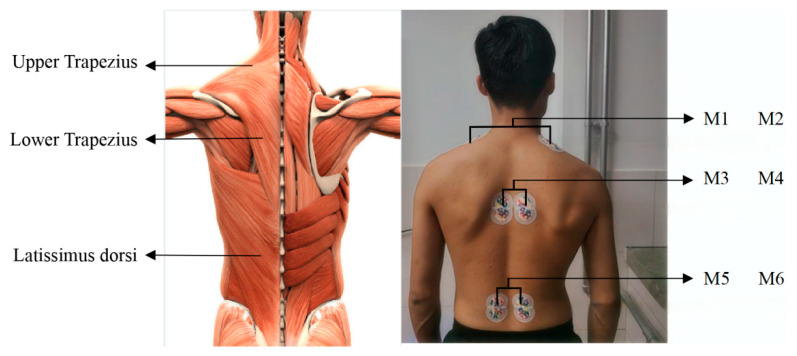
sEMG signals measured at the shoulder, back, and waist. A total of six sEMG signal acquisition points labeled from M1 to M6. Among them, M1, M3, and M5 correspond to muscles on the left side, while M2, M4, and M6 correspond to muscles on the right side.

The muscle fatigue of upper limb during load-bearing walking procedure was measured by surface EMG signals. Three pairs of electrodes were placed on the shoulder, back, and waist, respectively, as shown in Figure 3, recording the activities of the upper trapezius muscle, lower trapezium muscle, and latissimus dorsi.

To eliminate the impact of motion artifacts and respiration, wavelet threshold denoising (WTD) was adopted to process the EMG signal quickly. The WTD method has been demonstrated to be capable of preserving the nuances of biomedical signals whilst simultaneously attenuating extraneous noise, a feat accomplished by leveraging its multi-scale and multi-resolution characteristics [52]. Suppose Equation (1) represents a simple model of the EMG signal, where s(t) and n(t) denote sEMG signals and White Gaussian Noise $N(0,\sigma^{2})$, respectively.

(1) f(t)=s(t)+n(t)

The Daubechies 2 (db2) mother wavelet was selected for decomposition because its morphological structure closely resembles the typical shape of Motor Unit Action Potentials. The signal was decomposed to the 4th level, which effectively isolates the dominant frequency band of muscle activation (20–500 Hz) from high-frequency noise and low-frequency artifacts. Log Scale Modified Universal (LSMU) was adopted as the threshold $\lambda_{i}$ estimation method.

(2) $$\lambda_{i}=\sigma_{j}\sqrt{2\log\left(N\right)}/\log_{2}\left(j+1\right)$$

where N is the length in samples of time-domain signal and $\sigma_{j}$ is standard deviation at scale j (1–4).

Then, high-frequency detail coefficient $d_{j,k}$ could be calculated as shown in Equation (3).

(3) $$d_{j,k}=\sum_{n}g_{n-2k}d_{j+1,n}$$

where $g_{n-2k}$ is the high pass filter, k=0,1,⋯,N−1.

After threshold values are determined, signal reconstruction can be performed using soft transformation.

(4) $${\hat{d}}_{j,k}=\left\{\begin{array}{c} sgn(d_{j,k})\cdot (\left|d_{j,k}\right|-\lambda_{i})\;\;\left|d_{j,k}\right|≧\lambda_{i}\\ 0\;\;\left|d_{j,k}\right|<\lambda_{i} \end{array}\right.$$

Following preprocessing, the integrated electromyography (iEMG) was computed from the rectified signal using MATLAB R2022a for subsequent analysis.

(5) $$iEMG=\frac{1}{T}\int_{0}^{T}\left|x_{rec}(t)\right|dt$$

where $x_{rec}(t)$ is the reconstructed signal in microvolts (μV), T is the time window of 60 s.

#### 2.4.2. Subjective Fatigue Evaluation

**Figure 4:**
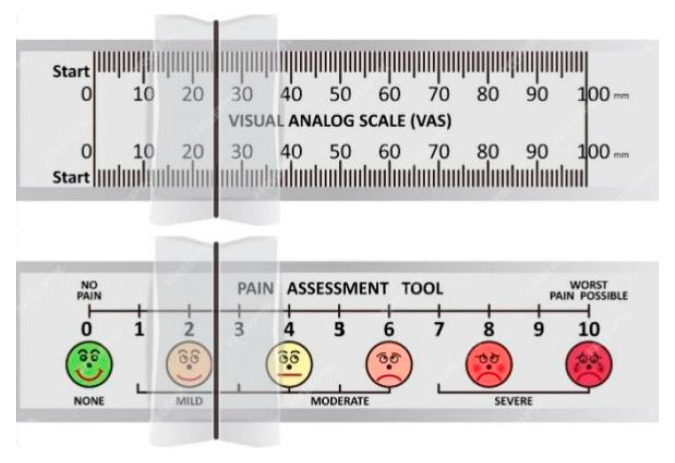
Visual Analog Scale (VAS) for measuring pain levels.

Participants performed subjective evaluations of overall and localized fatigue. An adapted version of a fatigue questionnaire for construction workers [53] was used to assess overall fatigue based on a 5-point scale, as shown in Appendix A. Localized muscular fatigue was assessed using a Visual Analog Scale, as shown in Figure 4. The scale consisted of a 100 mm horizontal line, with one end representing “no pain” and the other representing “worst pain”. Participants marked their perceived pain level on the line. During the experiment, they reported shoulder, back, and waist fatigue at five-minute intervals.

#### 2.4.3. Eye Metrics

Four eye movement metrics were recorded in this study: saccade times (ST), saccade speed (SS), saccade amplitude (SA), and pupil diameter (PD). A saccade is a rapid eye movement that shifts fixation to reposition the fovea for visual information acquisition. ST denotes the number of fixation shifts within a given period. SS represents the angular velocity of a saccadic movement (°/s), and SA refers to the angular displacement from the onset to the end of a saccade (°). PD represents the pupil size (mm), which controls retinal light intake and is sensitive to cognitive load and attentional effort. It is affected by illumination and is widely used as a psychophysiological indicator of neural activity [54].

To quantify changes in eye movement characteristics during load-walking, the average values of eye movement parameters during 60 s before and after the experiment were used as the baseline. The rate of change in eye movement parameters was calculated using Equation (6).

(6) $$Rate\;of\;Change\;in\;P=\frac{P_{t}-P_{0}}{P_{0}}$$

where t denotes the experimental time point in minutes. P denotes any eye movement metric, including ST, SS, SA, and PD. $P_{0}$ is the baseline value of the eye metric, $P_{0}$ represents the same parameters at time t.

To avoid interference from gaze fluctuations during subjective fatigue assessments, eye movement data from one minute before and after each self-rating were excluded. Only the three-minute interval between consecutive assessments was analyzed.

### 2.5. Experimental Procedure

All participants completed one experimental session comprising 12 trials. Tests were conducted between 14:00 and 16:00 in March 2024. Participants were instructed to consume a standardized meal 2 h before the experiment and to arrive fully hydrated. During the experiment, water was provided during rest intervals to prevent dehydration. Participants were instructed to avoid strenuous exercise and to strictly abstain from alcohol, caffeine, and performance-enhancing supplements for at least 24 h prior to the experiment.

**Figure 5:**
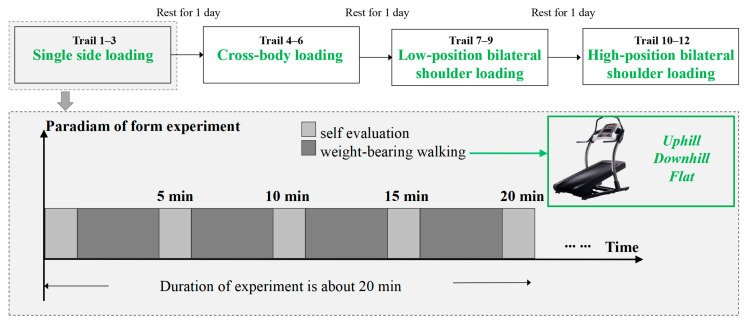
The experimental process of this study.

An overview of the experimental procedure is shown in Figure 5. To avoid cumulative fatigue, all sessions were completed within seven days, with one rest day between the test days and a 30 min break between the trials. The laboratory environment was controlled at 23 °C and 50% relative humidity, and all experimental instructions were given in advance. Surface EMG electrodes were applied to the participants, and eye-tracking calibration was completed prior to testing. Each trial consisted of 20 min of load-bearing walking, during which subjective fatigue was quickly assessed every five minutes while EMG signals and eye movement data were continuously recorded.

### 2.6. Statistical Analysis

Statistical analyses were performed using SPSS 27.0. As the samples met the assumptions of normality and homogeneity of variance across groups, analysis of variance (ANOVA) was applied. When significant differences were identified, the Bonferroni post hoc test was conducted to determine pairwise group differences. The level of statistical significance was set at *α* < 0.05. Pearson correlation analysis was conducted to evaluate the linear relationships among parameters. For each correlation, both the correlation coefficient ($R^{2}$) and its statistical significance were reported. Subsequently, multiple quadratic regression analyses were performed to model the association between subjective fatigue and objective parameters. Only predictors with statistical significance (*p* < 0.05) were retained in the final regression models.

## 3. Results

### 3.1. Change in iEMG Activity (**H1**)

#### 3.1.1. Effect of Loading Type on iEMG Activity

**Figure 6:**
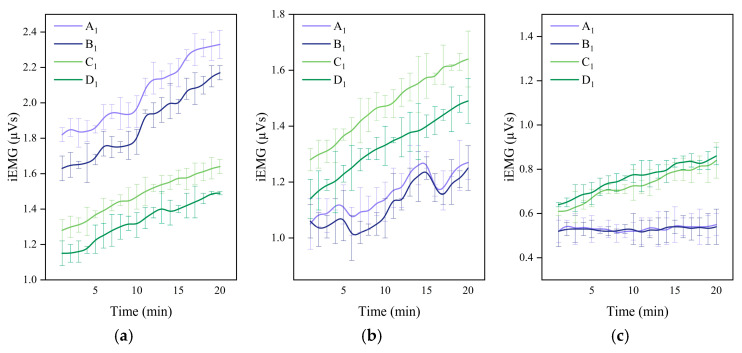

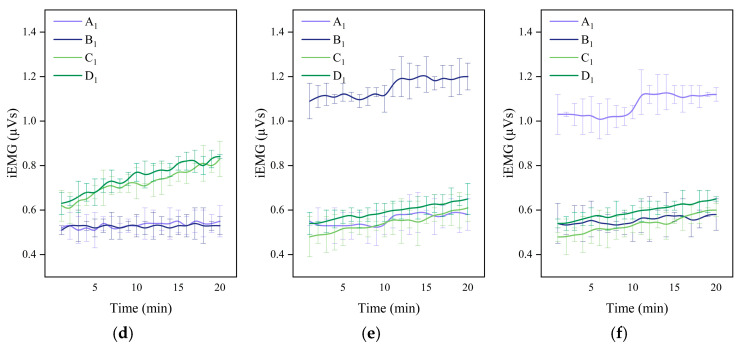
Temporal iEMG intensity under different loading types on the flat ground. (**a**) M1. (**b**) M2. (**c**) M3. (**d**) M4. (**e**) M5. (**f**) M6. Note: For statistical analysis of significant differences regarding loading types and road slopes, please refer to Figure 7 (Table 3) and Figure 8 (Table 4), respectively.

Figure 6 shows the temporal changes in iEMG intensity under different loading types. For asymmetric loading types (A and B), the weight was on the left shoulder. Accordingly, the shoulder–back–lumbar muscles are represented as M1–M3–M5 for the left side and M2–M4–M6 for the right side.

As shown in Figure 6 and Table 2, under single-sided loading (Type A), fatigue in the left upper trapezius (M1) increased rapidly from 1.8 to 2.3 μVs within 20 min, accompanied by compensatory activation of the contralateral lumbar erector spinae (M6, 1.09 ± 0.05 μVs). In contrast, the ipsilateral lower trapezius (M3) and lumbar muscles (M5) showed lower activation as 0.55 μVs. Under cross-shoulder loading (Type B), the ipsilateral lumbar erector spinae (M5) remained highly activated (1.18 ± 0.05 μVs), approximately 2.0–2.2 times that of M6, while the back muscles (M3 and M4) maintained low iEMG levels as 0.54 μVs. In comparison, the bilateral shoulder load type (C and D) exhibited better spatial balance in iEMG signals, with muscle fatigue primarily occurring in the trapezius muscles. The average iEMG signal of M3 and M4 reached 0.72 μVs, approximately 33% higher than load-bearing type A and B. While the waist exerted less force, with iEMG signals around 0.5 μVs.

**Figure 7:**
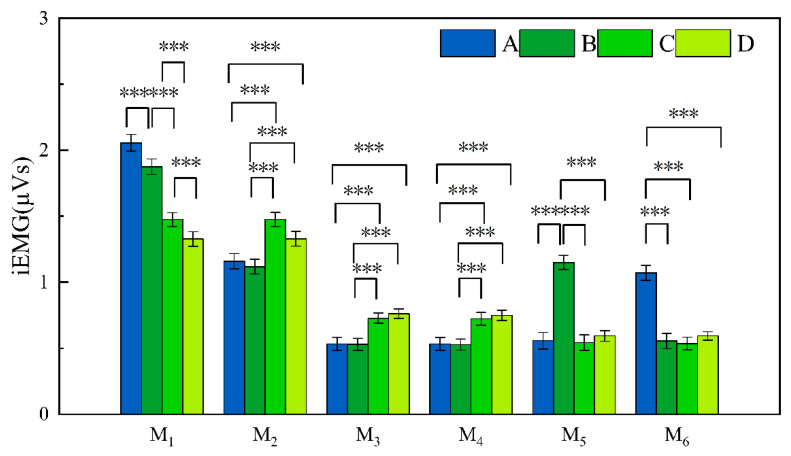
Comparison of iEMG across six muscle sites among four bearing methods. Note: *** *p* < 0.001.

As shown in Table 3, loading types significantly affected iEMG activity in all measured muscles (*p* < 0.001). Post hoc analyses with Bonferroni correction showed that (Figure 7), (1) for M1, fatigue was highest in Type A, followed by Type B, Type C, and lowest in Type D. (2) For M2–M4, Type C and D produced significantly greater fatigue than Type A and B. (3) For M5, Type B resulted in significantly higher fatigue than all other types, whereas for M6, Type A produced the highest fatigue compared to Types B–D. Detailed results are shown in Figure 7. These results support **H1** (a).

#### 3.1.2. Effect of Road Slope on iEMG Activity

**Figure 8:**
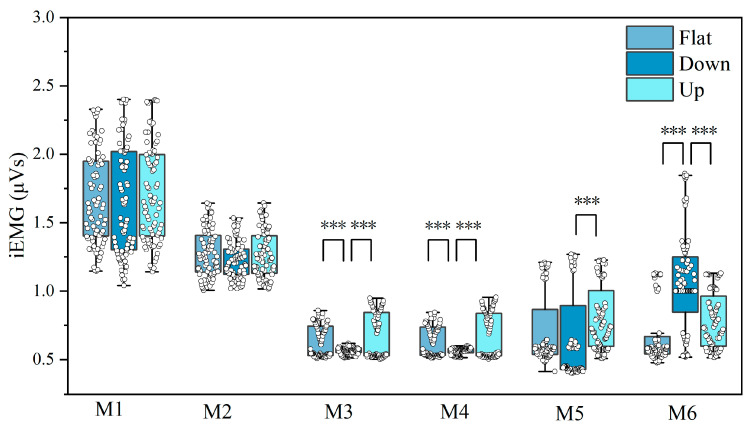
Variance of iEMG signal intensity of under different road slopes. Each box indicates the distribution range, and the white dots represent the mean iEMG values. Note: *** *p* < 0.001.

Figure 8 and Table 4 show that road slope significantly affected muscle activity in M3, M4, M5, and M6 (*p* < 0.01). Bonferroni post hoc analysis indicated that, (1) for M3 and M4, uphill walking produced significantly higher fatigue than flat or downhill (*p* < 0.001). (2) For M5, uphill walking caused significantly higher fatigue than downhill (*p* < 0.01). (3) For M6, downhill resulted in significantly higher fatigue than flat or uphill (*p* < 0.001). These results support **H1**(b).

### 3.2. Changes in Eye Metrics (**H2**)

#### 3.2.1. Rate of Change in ST, SS and SA

**Figure 9:**
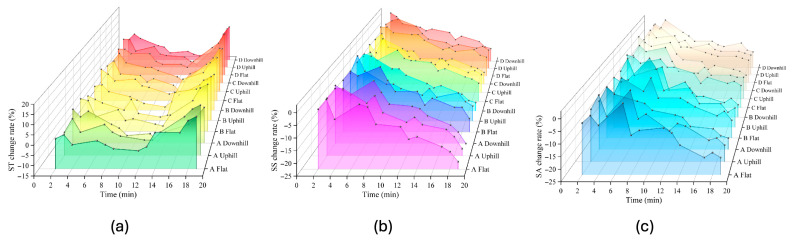
Temporal changes in eye movement parameters. (**a**) ST, (**b**) SS, and (**c**) SA.

As shown in Figure 9, ST, SS, and SA exhibited distinct temporal trends under different load-bearing types and ground conditions (Table 5, Table 6 and Table 7). The ST change rate showed a “fluctuation-decline-rise” pattern, with the greatest decrease under the uphill condition (−4.22%). SS and SA both followed a “fluctuation-decline” trend, with SS decreasing most under uphill walking (−9.49%) and SA showing minor variation (around −7.6%). The difference in SS and SA change rates for asymmetric loading (Types A and B) was 1.36 times that of symmetric loading (Types C and D). Loading type and road slope showed no significant influence on eye movement in terms of ST, SS, and SA.

#### 3.2.2. Changes in Pupil Diameter

**Figure 10:**
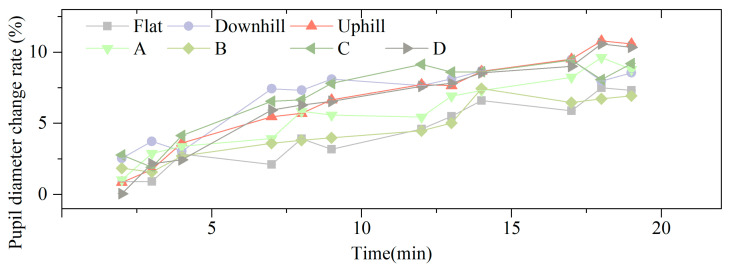
Temporal change in pupil diameter under different loading types and road slopes.

Figure 10 shows the temporal variation in pupil diameter relative to baseline. Overall, a “fluctuating-upward” trend was observed across all scenarios. Symmetric load types caused greater increases than asymmetric load types. For example, load types C and D exceeded 6.00%, compared to 5.75% and 4.55% for types A and B. Regarding road slope, downhill conditions produced the highest average increase (6.89%), followed by uphill (6.59%) and flat ground (4.27%).

Table 8 presents the effects of load type and road slope on pupil diameter. Load type significantly affected pupil diameter (*F* = 4.25, *p* = 0.007). Bonferroni post hoc results showed that the increase under cross-body loading was significantly lower than that under high-position (*p* = 0.006) and low-position (*p* = 0.049) bilateral shoulder loading. Road slope also had a significant effect (*F* = 10.99, *p* < 0.001). Flat ground produced significantly lower increases than uphill (*p* < 0.001) and downhill (*p* = 0.001) conditions. Loading type and road slope showed significant influences on eye movement in terms of pupil diameter. Therefore, the results support **H2** (a,b).

### 3.3. Changes in Self-Evaluation (H3)

#### 3.3.1. Visual Analog Scale (VAS)

Table 9 presents the VAS on the shoulder, back, and waist under different loading types and road conditions. Under single-side loading, the VAS on the shoulder was 6.23 ± 1.09, which was 1.83 times higher than the VAS on the back (3.41 ± 0.94) and 1.95 times higher than the VAS on the waist (3.19 ± 1.41). With cross-body loading, the VAS on the shoulder, back, and waist decreased by 20.55%, 26.69%, and 14.11%, respectively, compared with single-side loading, indicating that participants perceived greater local fatigue under single-side loading. Under bilateral-shoulder loading, the VAS on the shoulder was approximately 65% of that under asymmetric loading, while the VAS on the back and waist were 90% and 94%, respectively. Regarding road conditions, the VAS on the shoulder in the uphill condition (5.14 ± 1.69) was higher than that on the flat (4.51 ± 1.45) and downhill (4.86 ± 1.46) conditions. Similarly, the VAS on the back (3.58 ± 1.16) and the VAS on waist (3.78 ± 1.07) were higher during uphill walking compared with the flat and downhill conditions.

**Figure 11:**
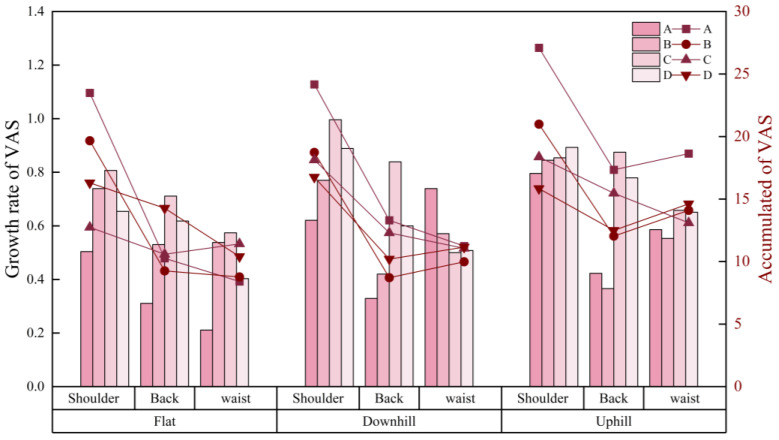
Growth rate and accumulation of VAS under all loading type and road slopes.

Figure 11 illustrates the VAS growth rate and cumulative VAS over time across different loading types and road slopes. The average growth rates for the four loading types were 0.502, 0.593, 0.757, and 0.666, with high-position bilateral shoulder loading showing the fastest fatigue increase (0.757) and single-side loading the slowest (0.502). In terms of cumulative VAS, the uphill condition yielded a higher total fatigue score (16.676) than flat (12.967) and downhill (13.812) conditions, although the difference was not statistically significant (*F* = 2.207, *p* = 0.126). Similarly, single-side loading resulted in the highest cumulative fatigue (17.107), but no significant difference was observed compared with the other loading types.

Table 10 summarizes the effects of load type and road slope on VAS scores over shoulder, back, and waist muscles. Load type significantly affected VAS on shoulder (*F* = 6.801, *p* = 0.001), while road slope significantly affected the VAS on the waist (*F* = 9.025, *p* = 0.001) and back (*F* = 3.267, *p* = 0.050). VAS on the shoulder under Type A was 6.23 ± 1.10, significantly higher than under Type B, C, and D, with values of 4.95 ± 1.30, 4.10 ± 1.51, and 4.08 ± 1.39. Under uphill conditions, the VAS on the back reached 3.59 ± 1.06, higher than under flat and downhill conditions, both at 2.78 ± 0.93. The VAS on the waist under uphill conditions was 4.53 ± 1.03, compared with 2.44 ± 0.74 on flat ground and 2.72 ± 0.98 under downhill conditions.

#### 3.3.2. Overall Fatigue Assessment

**Figure 12:**
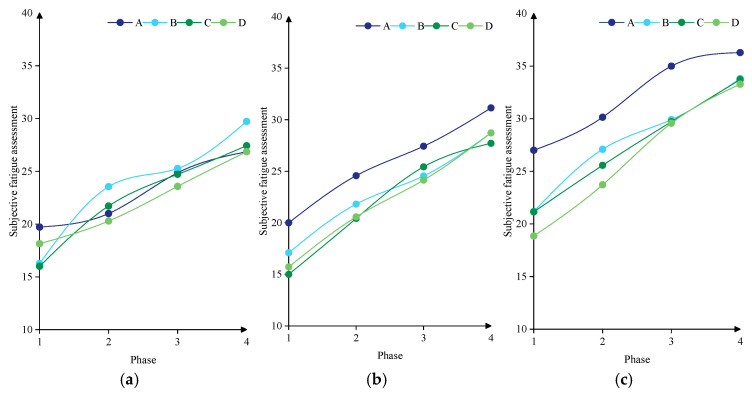
Variances of subjective fatigue assessment. (**a**) Flat. (**b**) Downhill. (**c**) Uphill.

In addition to local fatigue assessed by the VAS, overall fatigue was evaluated, as shown in Figure 12. Overall fatigue under uphill conditions ranged from 18.85 to 36.28, higher than under flat (16.00–29.73) and downhill conditions (15.00–31.14). The mean overall fatigue scores for load types A–D were 27.00, 24.91, 24.04, and 23.62, respectively. Table 11 summarizes the effects of load type and road slope on overall fatigue. Load type showed no significant effect (*F* = 1.064, *p* = 0.375), whereas road slope had a significant impact (*F* = 6.149, *p* = 0.005). Post hoc comparisons indicated that overall fatigue under uphill conditions (28.50 ± 5.35) was significantly higher than under flat (22.87 ± 4.41) and downhill conditions (23.32 ± 5.18). These results support **H3** (a,b).

### 3.4. Phases-Dependent Variations in Eye Movement Parameters (**H4**)

To further analyze eye movement signals, the collected data was divided into three temporal phases according to the progression of perceptual fatigue using the method in Fang et al.’s work [55]. They classified construction work procedures into three phases based on self-reported fatigue scores: <20, 20–30, and >30. Correspondingly, the three phases of our data were defined as 0–5 min (scores < 20), 6–14 min (scores 20–30), and 15–20 min (scores > 30).

**Figure 13:**
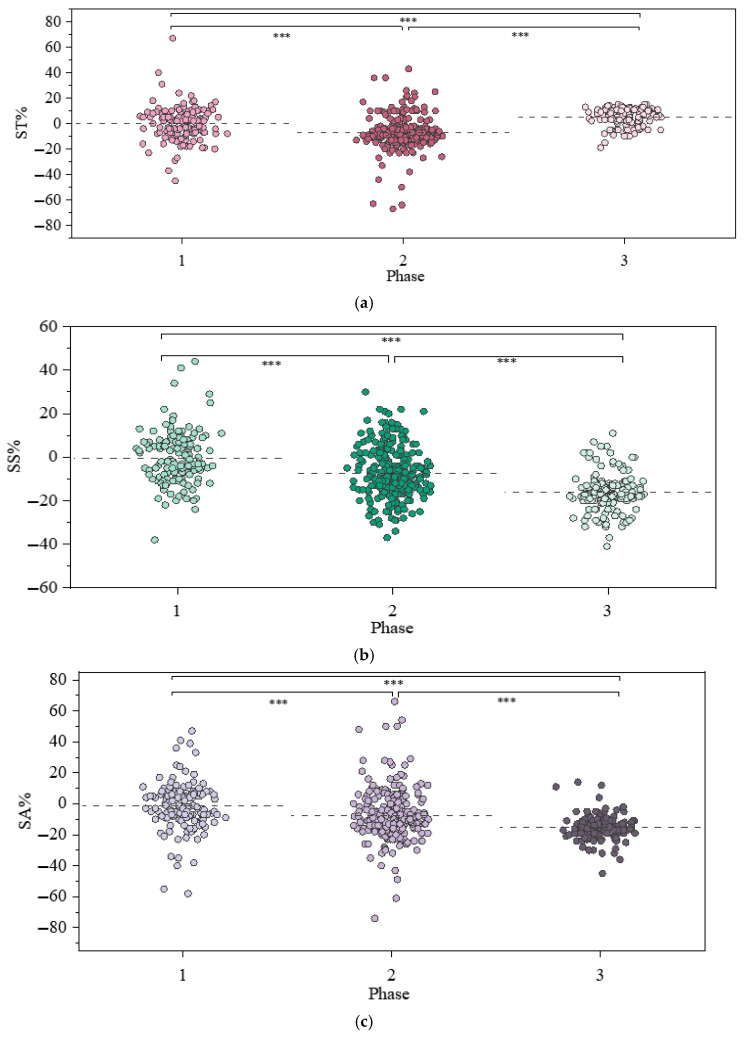
Statistics differences in eye movement parameter change rates across three experimental stages. (**a**) ST. (**b**) SS (**c**) SA. Note: *** *p* < 0.001.

To test H4, descriptive and statistical analyses were conducted; see the results in Figure 13 and Table 12. Descriptively, the rates of change in ST, SS, and SA slightly declined during Phase 1 (−0.16%, −0.58%, −1.32%), followed by a further decrease in Phase 2 (−7.33%, −7.31%, −7.39%). In Phase 3, ST exhibited a rebound to an average of 5.23%, while SS and SA continued to decline (−15.96%, −15.27%), indicating a “decline-increase” pattern only for ST.

Statistical analysis showed that rates of change in ST, SS, and SA differed significantly across the three stages (*p* < 0.001). Post hoc tests conducted with the Bonferroni correction indicated that the ST change rate was significantly higher in stage 1 than in stage 2 (*p* < 0.001) and higher in stage 3 than in both stage 1 (*p* < 0.001) and stage 2 (*p* < 0.001). For both SS and SA, change rates were significantly greater in stage 1 than in stages 2 and 3 and greater in stage 2 than in stage 3 (all *p* < 0.001). These results support **H4**.

**Figure 14:**
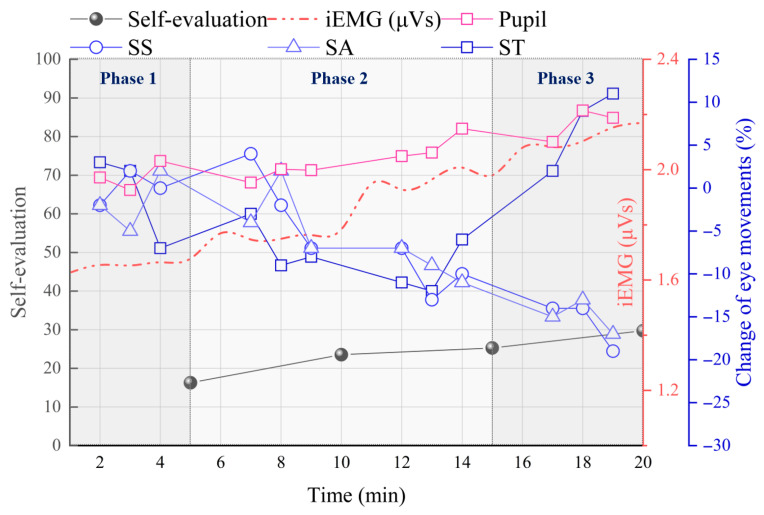
Temporal changes in surface EMG, self-evaluated fatigue, and eye movement parameters.

Figure 14 illustrates the temporal changes in multiple parameters across the three phases. While self-evaluated fatigue, EMG, and pupil size continuously increased, SS and SA showed a progressive decline. ST exhibited a sharp rise in Phase 3 following a steady decrease during Phases 1 and 2.

### 3.5. Correlations Among Multi-Dimensional Parameters (**H5**, **H6**)

**Figure 15:**
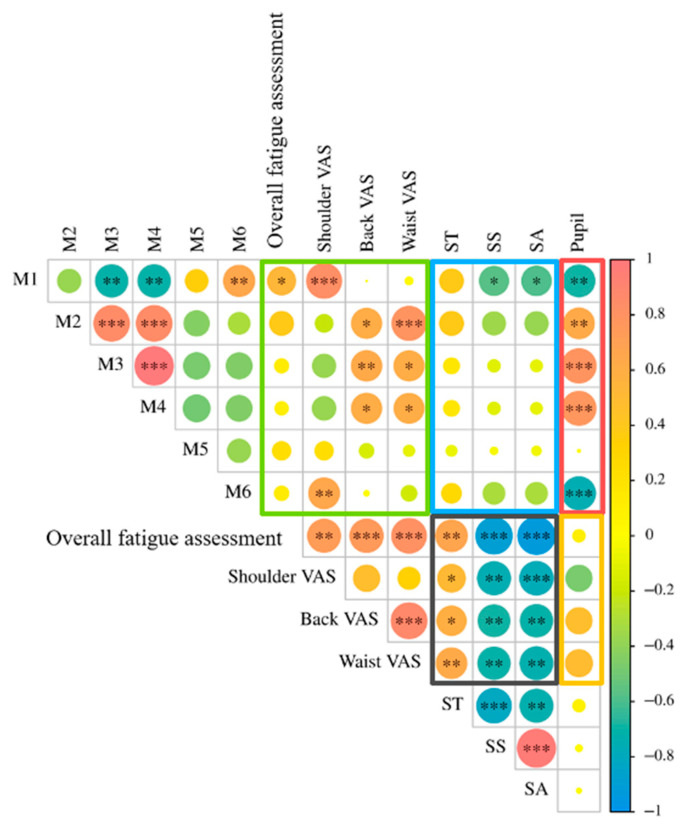
Pearson’s correlation coefficient among surface EMG, self-evaluated fatigue, and eye movement parameters. Note: * *p* < 0.05, ** *p* < 0.01, and *** *p* < 0.001.

Figure 15 presents the correlations among EMG signals (M1–M6), subjective fatigue (VAS and overall fatigue), and eye metrics (ST, SS, SA, and pupil diameter). Strong positive correlations were observed among M2, M3, and M4 (*R* > 0.88, *p* < 0.001). M1 was negatively correlated with M3 and M4, whereas M5 and M6 showed weak associations with other muscles. For subjective fatigue, the VAS on the back and waist were highly correlated (*R* = 0.94, *p* < 0.001), while the shoulder on the VAS was relatively independent. EMG signals of the shoulder and back were positively correlated with their corresponding VAS scores (*R* > 0.68, *p* < 0.01). Among the eye metrics, SS and SA were strongly positively correlated (*R* = 0.99, *p* < 0.001), and both were negatively correlated with ST. Pupil diameter showed no significant correlation with other eye metrics.

The saccade parameters, including ST, SS and SA, demonstrated a strong correlation with subjective fatigue (average *R* = −0.77); see the results in the black box in Figure 15. However, pupil diameter showed only a moderate correlation with subjective ratings (average *R* = −0.46); see the results in the yellow box in Figure 15. Therefore, the results support H5.

Pupil diameter showed a strong correlation with EMG signals (average *R* = −0.71); see the results in the red box in Figure 15. The saccadic parameters (ST, SS, SA) exhibited a weak correlation with EMG (average *R* = −0.23); see the results in the blue box in Figure 15. Therefore, the results support H6.

To answer RQ2, while EMG signals are capable of characterizing physiological muscle fatigue, their correlation with subjective fatigue ratings was found to be weak, as highlighted by the green box in Figure 15 (average *R* = −0.22). In contrast, saccade parameters, including ST, SS, and SA, demonstrated a strong correlation with subjective fatigue (black box). So, eye movement metrics showed their ability to characterize physical fatigue. Meanwhile, eye movement metrics that are highly correlated with EMG signals (i.e., pupil diameter, red box) showed a moderate to weak correlation with subjective measures (yellow box), whereas eye movement metrics showing only moderate correlations with EMG (i.e., saccade parameters, blue box) showed strong correlation with the subjective measures (black box). Overall, to better represent physical fatigue quantitatively, we incorporated eye tracking metrics into the assessment model (Section 3.6).

### 3.6. Multilateral Quadratic Regression Analysis

To answer RQ3, a multilateral quadratic regression analysis was conducted on the subjective assessment and objective parameters, retaining only those with significance (*p* < 0.05). A fitting equation was shown in Table 13, to model the relationship among five variables, where dependent variable is the overall fatigue assessment (Y), independent variables contain iEMG ($X_{1}$) and eye movement parameters ($X_{2}$: ST, $X_{3}$: SS, $X_{4}$: SA). It should be noted that pupil diameter exhibited relatively weak correlations with the subjective fatigue assessments; hence, they were excluded from the regression analysis. Related coefficients across the four of load types and three road slopes showed strong correlation ($R^{2}$ ≥ 0.90). A leave-one-out cross-validation (LOOCV) approach was adopted to assess model generalizability. The root mean square error (RMSE) values ranged from 0.19 to 0.94, which are substantially lower than 10% of the measurement range. The overall fatigue assessment was mainly related to the iEMG, SS change rate, and SA change rage, while the ST change rate did not play a significant role.

## 4. Discussion

In this study, we empirically investigated changes on SEM, eye movement, and perceptual metrics during load-bearing walking, with a focus on eye movement parameters. Four loading types and three road slope conditions were tested to simulate the operational tasks of emergency rescuers. The results indicate that eye movement metrics, including saccade parameters and pupil diameter, can serve as non-invasive indicators for revealing physical fatigue when combined with EMG metrics.

### 4.1. Effect of Loading Type on Electromyography Signals

Our experiment showed that single-sided loading induced greater muscle fatigue in the contralateral waist, whereas cross-body loading primarily stimulated muscle fatigue in the ipsilateral waist. These findings are consistent with the asymmetrical EMG activity reported in previous studies [5,6]. A possible explanation is that the waist muscles play a critical role in providing the counterbalancing moments necessary to maintain an upright posture during load-bearing walking [7]. The results also indicate that a symmetrical backpack configuration provided greater stability and results in lower regional discomfort. In addition, EMG activity in the upper trapezius muscles was higher under high-position shoulder loading compared to low-position loading. This increase may be attributed to compensatory activation of the trapezius muscles. To maintain walking stability under higher load placement, such compensatory activation leads to elevated EMG levels in the trapezius, as also reported in previous studies [56,57].

### 4.2. Effect of Road Slope on Muscle Activation Patterns

Our experiment revealed that uphill conditions led to greater waist muscle activity compared with downhill conditions. This can be explained by a backward shift in the combined center of gravity of the trunk and backpack, which induces a compensatory forward trunk lean [10]. As a result of this counterbalancing mechanism during uphill walking, waist muscle activity is reduced under downhill conditions [8]. During downhill walking, backward movements of the trunk are required to shift the center of mass posteriorly, thereby reducing the forward momentum induced by gravity [9].

### 4.3. Changes in Pupil Diameter During Load-Bearing Walking

In current study, we found that pupil diameter initially fluctuates and continuously increases during the execution of the load-bearing walking. The similar trend has been confirmed in previous studies [58,59]. It may be explained by that pupil size reflects the level of effort invested in a task, whether physical or mental in nature [60]. However, the opposite patterns were found in other studies [61,62], in which pupil diameter decreased when participants engaged in tasks requiring sitting or standing. This discrepancy may stem from differences in task characteristics and experimental conditions [62]. The size of the pupil is jointly regulated by the sympathetic nerve and the parasympathetic nerve of the autonomic nervous system. Various tasks and experiment durations have different impacts on these two pathways [63,64]. We also identified that the pupil diameter is relatively larger when subjects walked on the inclined surfaces rather than flat surfaces. This may be explained by the combined contributions of the visual and vestibular systems to postural orientation and balance during inclined walking [65].

### 4.4. Saccade Metrics as Fatigue Indicators During Load-Bearing Walking

Our observations also showed that the rates of change in saccade speed (SS) and saccade amplitude (SA) exhibited a progressive decline over time, whereas saccade time (ST) followed a fluctuation–decline–rise pattern. These findings are consistent with previous studies on mental fatigue [66,67,68], suggesting a shared neural substrate underlying both mental and physical exhaustion. Saccade generation is primarily controlled by the superior colliculus and brainstem [69]. Lesions in omnipause neurons have been associated with reductions in saccade velocity, resembling the effects observed under fatigue conditions [70]. Overall, our findings challenge the traditional dichotomy between physical and mental fatigue by demonstrating that load-bearing walking imposes a substantial neural cost, manifested as a progressive decline in saccade-related metrics.

### 4.5. Multidimensional Fatigue Evaluation

Current approaches to fatigue assessment typically rely on multi-parameter models incorporating EEG, ECG, PPG, and EOG signals [28,29,30,34,35,36,37,38,71]. A critical gap remains in the application of oculomotor metrics for detecting physical fatigue, particularly in dynamic load-bearing scenarios where several sensors are impractical due to motion artifacts and discomfort. To bridge this gap, our study established a multilateral regression model integrating eye-tracking metrics and EMG signals. This integration is significant for two reasons. First, while EMG captures peripheral muscle failure, eye metrics provide a unique lens into the participant’s perceived effort. Second, the high correlation coefficients demonstrate that eye-tracking parameters are robust predictors of physical fatigue.

### 4.6. Limitation and Future Work

First, it is important to acknowledge that the current experiment did not distinguish physical fatigue versus central cognitive fatigue on eye movement parameters. Instead, the variations in pupil diameter and saccade metrics reported should be interpreted as a reflection of the systemic load, a composite of the physiological and cognitive demands imposed on the nervous system during load-bearing walking. Second, we recognized that the 100 Hz sampling rate of the eye-tracking glasses, while validated for analyzing macro-temporal trends in fatigue development, fell below the gold standard for micro-saccadic analysis. Future investigations aiming to characterize subtle kinematic features should employ tracking devices with higher sampling frequencies.

Regarding future work, to enhance the validity of our findings, future work could focus on three areas. First, testing duration will be expanded to better simulate the prolonged intensity of emergency rescue missions. Second, the participant pool will be diversified to include field personnel, bridging the gap between laboratory data and real-world application. Third, higher-dimensional eye-tracking features, multi-site EMG data, and facial feature recognition will be integrated to construct a more robust, multi-feature fatigue monitoring framework, improving safety and performance in load-bearing environments.

## 5. Conclusions

Emergency rescue personnel are often required to carry heavy equipment for extended periods during both training and operational tasks. Consequently, musculoskeletal disorders have become a major occupational health concern among rescuers. To address this issue, this study proposes a multidimensional fatigue evaluation framework that integrates eye-tracking metrics and EMG signals to facilitate physical fatigue monitoring in load-bearing walking scenarios. The main findings are summarized as follows:

(1) **H1(a,b):** Loading type significantly influenced iEMG activity in all shoulder-back-waist muscles (*p* < 0.001). Additionally, road slope significantly influenced back and waist muscle activity (*p* < 0.01).
(2) **H2(a,b):** Loading type and road slope showed no significant influence on eye movement in terms of ST, SS, and SA. The rate of change in saccade time (ST) followed a “fluctuation-decline-rise” trend, while saccadic speed (SS) and amplitude (SA) exhibited a “fluctuation-decline” pattern. Load type and road slope showed significant effects on pupil diameter (*F* = 4.25, *p* = 0.007; *F* = 10.99, *p* < 0.001).
(3) **H3(a,b):** Loading type significantly affected shoulder fatigue ratings (*F* = 6.801, *p* = 0.001), while slope significantly influenced waist (*F* = 9.025, *p* = 0.001) and back fatigue (*F* = 3.267, *p* = 0.050).
(4) **RQ1:** Loading type and road slope significantly influenced people’s EMG, eye movement, and perceptual responses during load-bearing walking. This indicates that physical fatigue can be assessed and represented by EMG and eye movement metrics.
(5) **H4:** Significant differences were observed in saccade parameters across the three stages, defined by perceptual fatigue (*p* < 0.001). ST rebounded in Phase 3 (+2.61%), whereas SS and SA continued to decline (−14.83%, −14.52%).
(6) **H5:** Saccade parameters, including ST, SS, and SA, demonstrated a strong correlation with subjective fatigue (average R = −0.77). However, pupil diameter only showed a moderate correlation with subjective ratings (average R = −0.46).
(7) **H6:** Pupil diameter showed a strong correlation with EMG signals (average R = −0.71), while the saccadic parameters (ST, SS, SA) exhibited a weak correlation with EMG (average R = −0.23).
(8) **RQ2:** Eye movement signals showed the potential to reveal physical fatigue. More specifically, eye movement metrics that are highly correlated with EMG signals (i.e., pupil diameter) did not effectively characterize the subjective measures, whereas eye movement metrics showing only moderate correlations with EMG (i.e., saccade parameters) showed a strong correlation with the subjective measures. This indicates that eye movement metrics should play a role in the equation of assessing people’s physical fatigue.
(9) **RQ3:** A multilateral quadratic regression analysis was conducted on subjective assessment and objective parameters. Related coefficients across the four of load types and three road slopes showed strong correlation (R^2^ ≥ 0.90). This indicates that eye movement metrics can be integrated in the equation quantitatively to assess physical fatigue during load-bearing walking.

Overall, this study advances the theoretical understanding of physical fatigue and demonstrates the potential of eye movement metrics as non-invasive indicators for fatigue monitoring.

## Figures and Tables

**Table 1:** Independent variables (with levels) tested in this study.

Road Slope Conditions	Loading Types			
	Single-Sided Loading	Cross-Body Loading	Low-Position Bilateral Shoulder Loading	High-Position Bilateral Shoulder Loading
Flat	$A_{1}$	$B_{1}$	$C_{1}$	$D_{1}$
Downhill	$A_{2}$	$B_{2}$	$C_{2}$	$D_{2}$
Uphill	$A_{3}$	$B_{3}$	$C_{3}$	$D_{3}$

**Table 2:** Variation in iEMG amplitude and lateral asymmetry of shoulder–back–waist muscles.

Load Types	Part	iEMG		*F*	*p*
		Left	Right		
A	Shoulder	2.11 ± 0.19	1.16 ± 0.07	1279.954	<0.001 ***
B	Shoulder	1.88 ± 0.18	1.12 ± 0.08	890.587	<0.001 ***
C	Shoulder	1.45 ± 0.12	1.44 ± 0.12	0.004	0.952
D	Shoulder	1.30 ± 0.12	1.30 ± 0.11	0.008	0.931
A	Back	0.53 ± 0.01	0.55 ± 0.03	0.048	0.827
B	Back	0.55 ± 0.03	0.53 ± 0.01	0.593	0.442
C	Back	0.71 ± 0.12	0.70 ± 0.11	0.048	0.827
D	Back	0.72 ± 0.13	0.72 ± 0.13	0.002	0.967
A	Waist	0.57 ± 0.04	1.09 ± 0.05	3801.026	<0.001 ***
B	Waist	1.18 ± 0.05	0.57 ± 0.04	5654.716	<0.001 ***
C	Waist	0.56 ± 0.11	0.56 ± 0.11	0.007	0.935
D	Waist	0.60 ± 0.17	0.60 ± 0.18	0.008	0.929

Note: *** *p* < 0.001.

**Table 3:** Variation in iEMG amplitude under four load-bearing types.

Muscle	Load-Bearing Types				*F*	*p*
	A	B	C	D		
M1	2.11 ± 0.19	1.88 ± 0.18	1.45 ± 0.12	1.30 ± 0.12	350.739	<0.001 ***
M2	1.16 ± 0.07	1.12 ± 0.08	1.44 ± 0.12	1.30 ± 0.11	130.986	<0.001 ***
M3	0.53 ± 0.01	0.55 ± 0.03	0.71 ± 0.12	0.72 ± 0.13	78.975	<0.001 ***
M4	0.55 ± 0.03	0.53 ± 0.01	0.70 ± 0.11	0.72 ± 0.13	81.843	<0.001 ***
M5	0.57 ± 0.04	1.18 ± 0.05	0.56 ± 0.11	0.60 ± 0.17	473.019	<0.001 ***
M6	1.09 ± 0.05	0.57 ± 0.04	0.56 ± 0.11	0.60 ± 0.18	329.649	<0.001 ***

Note: *** *p* < 0.001.

**Table 4:** Variation in iEMG amplitude under three road slopes.

Muscle	Road Conditions			*F*	*p*
	Flat	Downhill	Uphill		
M1	1.68 ± 0.33	1.66 ± 0.40	1.70 ± 0.35	0.369	0.692
M2	1.27 ± 0.17	1.23 ± 0.14	1.27 ± 0.17	2.078	0.127
M3	0.64 ± 0.12	0.57 ± 0.03	0.68 ± 0.16	19.349	<0.001 ***
M4	0.63 ± 0.11	0.56 ± 0.02	0.68 ± 0.16	21.318	<0.001 ***
M5	0.71 ± 0.26	0.67 ± 0.32	0.80 ± 0.23	5.045	0.007
M6	0.69 ± 0.23	0.78 ± 0.20	0.65 ± 0.29	41.001	<0.001 ***

Note: *** *p* < 0.001.

**Table 5:** Change rate of saccade time (ST) among different ground conditions and bearing conditions.

Bearing Condition	Characteristic Value	Ground Condition		
		Flat	Downhill	Uphill
Single side loading	M ± SD	0.41 ± 0.52	−0.20 ± 0.60	−4.44 ± 0.60
	Maximum difference	17.45	18.65	18.08
Cross-body loading	M ± SD	−2.46 ± 0.79	−2.71 ± 0.75	−4.45 ± 0.58
	Maximum difference	23.71	23.20	18.19
High-position bilateral shoulder loading	M ± SD	−1.26 ± 0.69	−2.73 ± 0.78	−3.79 ± 0.62
	Maximum difference	22.05	26.70	20.57
Low-position bilateral shoulder loading	M ± SD	−1.31 ± 0.66	−2.35 ± 0.70	−4.18 ± 0.66
	Maximum difference	22.25	23.76	20.51

**Table 6:** Change rate of saccade time (SS) among different ground conditions and bearing conditions.

Bearing Condition	Characteristic Value	Ground Condition		
		Flat	Downhill	Uphill
Single side loading	M ± SD	−8.1 ± 0.81	−7.35 ± 0.80	−9.13 ± 0.82
	Maximum difference	24.75	25.07	27.20
Cross-body loading	M ± SD	−6.71 ± 0.73	−9.08 ± 0.66	−9.63 ± 0.78
	Maximum difference	23.45	23.20	23.09
High-position bilateral shoulder loading	M ± SD	−7.18 ± 0.52	−8.24 ± 0.575	−10.29 ± 0.58
	Maximum difference	15.90	18.70	19.76
Low-position bilateral shoulder loading	M ± SD	−7.12 ± 0.58	−7.85 ± 0.64	−8.90 ± 0.62
	Maximum difference	15.95	17.40	19.96

**Table 7:** Change rate of saccade time (SA) among different ground conditions and bearing conditions.

Bearing Condition	Characteristic Value	Ground Condition		
		Flat	Downhill	Uphill
Single side loading	M ± SD	−8.38 ± 0.68	−7.79 ± 0.71	−9.08 ± 0.72
	Maximum difference	22.19	22.81	21.77
Cross-body loading	M ± SD	−7.35 ± 0.62	−6.85 ± 0.77	−9.55 ± 0.77
	Maximum difference	19.38	25.42	22.88
High-position bilateral shoulder loading	M ± SD	−6.88 ± 0.54	−7.74 ± 0.61	−7.99 ± 0.67
	Maximum difference	14.88	15.97	19.49
Low-position bilateral shoulder loading	M ± SD	−6.66 ± 0.54	−7.65 ± 0.56	−8.55 ± 0.57
	Maximum difference	16.06	17.21	18.39

**Table 8:** Change rate of pupil diameter among different ground conditions and load types.

Factor	Condition	Value (%)	*F*	*p*
Loading type	A	5.75 ± 0.32	4.25	0.007 **
	B	4.55 ± 0.26		
	C	6.92 ± 0.33		
	D	6.45 ± 0.40		
Road slope	Flat	4.27 ± 0.26	10.99	<0.001 ***
	Down hill	6.89 ± 0.31		
	Uphill	6.59 ± 0.38		

Note: ** *p* < 0.01, and *** *p* < 0.001.

**Table 9:** VAS in the shoulder, back, and waist under different loading types and road slopes.

Road Slope	Body Part	VAS Value			
		Loading Type—A	Loading Type—B	Loading Type—C	Loading Type—D
Flat	Shoulder	5.87 ± 0.83	4.92 ± 1.21	3.19 ± 1.36	4.08 ± 1.09
	Back	2.57 ± 0.57	2.32 ± 0.91	2.65 ± 1.21	3.57 ± 1.02
	Waist	2.10 ± 0.35	2.19 ± 0.91	2.86 ± 1.01	2.60 ± 0.67
Downhill	Shoulder	6.04 ± 1.07	4.68 ± 1.62	4.53 ± 1.71	4.19 ± 1.54
	Back	3.32 ± 0.58	2.18 ± 0.69	3.08 ± 1.46	2.55 ± 0.10
	Waist	2.81 ± 1.24	2.50 ± 0.98	2.76 ± 0.85	2.79 ± 0.86
Uphill	Shoulder	6.75 ± 1.39	5.25 ± 1.41	5.25 ± 1.41	3.96 ± 1.53
	Back	4.33 ± 0.70	3.01 ± 0.61	3.87 ± 1.53	3.13 ± 1.38
	Waist	4.66 ± 1.01	3.52 ± 0.91	3.27 ± 1.10	3.66 ± 1.10

**Table 10:** Effects of load type and road slope on VAS over shoulder, back, and waist muscles.

Condition	Shoulder		Back		Waist	
	*F*	*p*	*F*	*p*	*F*	*p*
①	6.801	0.001	1.711	0.182	0.474	0.703
②	0.892	0.419	3.267	0.050	9.025	0.001

① Loading type. ② Road slope.

**Table 11:** Effect of Load-bearing method and road slope on subjective fatigue assessment.

Condition	Flat	Downhill	Uphill
A	23.11 ± 3.32	25.79 ± 4.70	32.11 ± 4.31
B	23.70 ± 5.60	23.05 ± 4.88	28.00 ± 5.32
C	22.46 ± 4.90	22.14 ± 5.65	27.54 ± 5.41
D	22.21 ± 3.82	22.29 ± 5.50	26.36 ± 6.37
Load-bearing method	*F* = 1.064, *p* = 0.375		
Road slope	*F* = 6.149, *p* = 0.005		

**Table 12:** Results of eye movement changes across the three phases.

Parameters	Phases			*F*	*p*
	1	2	3		
ST	−0.16 ± 1.29	−7.33 ± 1.21	5.23 ± 0.66	66.481	<0.001 **
SS	−0.58 ± 1.16	−7.31 ± 1.11	−15.96 ± 0.86	80.360	<0.001 **
SA	−1.32 ± 1.50	−7.39 ± 1.48	−15.27 ± 0.78	39.501	<0.001 **

Note: ** *p* < 0.01.

**Table 13:** Fitted regression equation between self-evaluated fatigue (Y), iEMG ($X_{1}$), ST ($X_{2}$), SS ($X_{3}$) and SA ($X_{4}$).

Condition	Regression Equation	$R^{2}$	RMSE
Load-bearing A	$Y=7.27X_{1}+0.34X_{2}-0.94X_{3}-0.91X_{4}-0.04X_{3}^{2}-0.03X_{4}^{2}+9.47$	0.90	0.54
Load-bearing B	$Y=11.03X_{1}-0.35X_{2}-1.15X_{3}+2.21X_{4}-0.18X_{3}^{2}+0.26X_{3}X_{4}+4.42$	0.91	0.94
Load-bearing C	$Y=27.56X_{1}-0.87X_{2}+1.28X_{4}-0.17X_{4}^{2}-0.04X_{2}X_{4}+0.09X_{3}X_{4}-13.42$	0.96	0.94
Load-bearing D	$Y=30.51X_{1}-0.34X_{3}+0.38X_{4}-0.10X_{3}^{2}+0.13X_{3}X_{4}-21.66$	0.96	0.30
Flat	$Y=0.02X_{1}-0.08X_{3}-1.44X_{4}-0.64X_{3}^{2}-0.70X_{4}^{2}+1.29X_{3}X_{4}+18.01$	0.91	0.57
Down	$Y=4.30X_{1}+0.41X_{2}-0.70X_{3}-0.57X_{4}+0.02X_{2}X_{4}-0.04X_{3}X_{4}+10.77$	0.92	0.78
Up	$Y=7.20X_{1}+0.33X_{2}-0.76X_{3}-1.01X_{4}+0.11X_{3}^{2}+0.13X_{4}^{2}-0.30X_{3}X_{4}+9.09$	0.91	0.19

## Data Availability

Processed datasets supporting the findings of this study will be made available on request.

## Appendix A

The overall fatigue assessment scale.

**Table:**

Question	Degree				
	Not at All	Slight	A Little Bit	Much	Completely
	1	2	3	4	5
Q1. Feel painful in the lower back					
Q2. Feel painful in the shoulder					
Q3. Feel painful in the upper back					
Q4. Whole body feel tired					
Q5. Have less strength in muscle					
Q6. Legs feel heavy					
Q7. My body movement slows down					
Q8. My thoughts easily wander					
Q9. Yawning					
Q10. Eyes feel strained					
